# Transcriptome analysis reveals genes associated with the bitter-sweet trait of apricot kernels

**DOI:** 10.48130/forres-0024-0004

**Published:** 2024-02-29

**Authors:** Yu Zhang, Wenquan Bao, Ta-na Wuyun, Mengzhen Huang, Chen Chen, Dun Ao, Rong Yang, Haiguang Huang, Lin Wang

**Affiliations:** 1 College of Forestry, Inner Mongolia Agricultural University, Hohhot 010018, China; 2 State Key Laboratory of Tree Genetics and Breeding, Research Institute of Non-Timber Forestry, Chinese Academy of Forestry, Zhengzhou 450003, China; 3 Key Laboratory of Non-Timber Forest Germplasm Enhancement & Utilization of National Forestry and Grassland Administration, Research Institute of Non-Timber Forestry, Chinese Academy of Forestry, Zhengzhou 450003, China; 4 College of Resources and Environmental Engineering, Shandong Agriculture and Engineering University, Jinan 250100, China; 5 Inner Mongolia Academy of Forestry, Hohhot 010010, China

**Keywords:** Apricot, Amygdalin, Prunasin, RNA-seq, Metabolic pathway

## Abstract

Prunasin and amygdalin are important factors that influence the kernel taste of apricots, however, the regulatory mechanisms underlying this are unclear. In this study, we analyzed the phenotype and transcriptome of kernels during development in *Prunus sibirica* (bitter kernels) and *Prunus armeniaca* × *Prunus sibirica* (kernel consumption apricot, sweet kernels). Prunasin and amygdalin content was significantly higher in bitter kernels compared with that in sweet kernels. Prunasin content exhibited a decreasing trend in both bitter and sweet kernels. The fastest decline was observed in bitter and sweet kernels during S3–S4 (82.21%) and S2–S3 (59.65%), respectively. The amygdalin content in the bitter kernels exhibited the fastest increase between 45–60 d after flowering, and reached a peak at 6.22% on 60 d after flowering. In contrast, the peak in sweet kernels occurred at 60 d after flowering, with a much lower content of 0.18%. Transcriptome analysis revealed 6,942 differentially expressed genes (DEGs), with a subset of 38 DEGs specifically enriched in the cyanoamino acid metabolic pathway. Among these, the ten candidate genes, including *CYP79*, *CYP71*, *UGT1*, *AH*, and *PH*, were identified as crucial in regulating prunasin and amygdalin metabolism. Furthermore, a weighted gene co-expression network analysis (WGCNA) unveiled two modules that exhibited significant correlation with prunasin and amygdalin content. Five DEGs were located at the center of the co-expression network, and were identified as hub genes, with four positively regulating prunasin content and one negatively regulating amygdalin content. Our results provide novel insights into the molecular-level regulation of the apricot kernel taste.

## Introduction

Apricot belongs to the genus *Armeniaca* in Rosaceae and is a deciduous perennial tree species that is primarily distributed in the 'Three North' regions of China, Russia, Siberia, and Mongolia as well as other places, and the germplasm resource is extremely rich^[1]^. Apricot kernels are full of nutrients, such as fat and protein. The unsaturated fatty acid content can reach 70%^[2]^ and the protein content can reach 25%^[3]^. The *Prunus armeniaca* × *Prunus sibirica* (kernel consumption apricot) is typically characterized by large and sweet kernels^[4]^, with extremely low amygdalin content (average 0.86%), and is mainly grown for kernel consumption in Northern China^[5]^. Wild *Prunus sibirica* (Siberian apricot) is small and practically inedible due to the kernels have a higher amygdalin content (average 5.56%)^[5]^, and *P. sibirica* kernels are useful medicinal components^[6]^. Therefore, apricot kernels with high amygdalin content for medicinal use and low amygdalin content for consumption are needed. It is important to cultivate apricot species with both low and high amygdalin content.

Amygdalin belongs to a group of aromatic cyanogenic glycosides and primarily consists of two components: glucose and amygdalinitrile^[7,8]^. Amygdalin is commonly found in the seeds of Rosaceae plants, such as *P. sibirica* (4%–6%)^[9]^, *Prunus salicina* (< 1.78%), and *Prunus persica* (< 0.68%)^[10]^. With respect to medicinal use, amygdalin exhibits anti-inflammatory, cough suppressant, and immunomodulatory activities^[11−13]^. It has a unique kernel odor and may be used in the food and cosmetics industries^[14]^. However, amygdalin metabolites (i.e., HCN) are potentially toxic, which limits their value. Therefore, reducing their bitterness is an important step in processing^[15]^.

The metabolic processes of prunasin and amygdalin may be divided into three parts: synthesis, degradation, and detoxification^[16]^. The synthesis of amygdalin begins with phenylalanine, which is converted into monocrotaline by two cytochrome *P450* enzymes (*CYP79* and *CYP71*) and a UDP-glucosyltransferase (*UGT1*). Monocrotaline is converted to amygdalin by another UDP-glucosyltransferase (*UGT2*). The process of amygdalin degradation primarily involves two β-glucosidases (*AH* and *PH*), which hydrolyze amygdalin along with lentilene lyase (*MDL1*). There are two pathways for the detoxification process of amygdalin. The first is the synthesis of β-cyanoalanine from HCN and L-cysteine, which is catalyzed by L-3-cyanoalanine synthetase (*CAS*). The second is the production of L-aspartic acid and L-asparagine and ammonia from β-cyanoalanine, which is catalyzed by the bifunctional nitrile hydratase *NIT4*^[17]^. Currently, the regulation of amygdalin metabolism in apricot kernels is unknown and requires further study.

Most studies on amygdalin have involved extraction^[18,19]^, content determination^[20]^, and biological activity^[21−23]^. Few studies have identified the genes associated with amygdalin formation and the early selection of the bitter traits of apricot kernels. Therefore, it is important to study the synthesis and regulation mechanisms of amygdalin. In the present study, we measured prunasin and amygdalin content, analyzed changes in their accumulation, and performed transcriptional profiling at different developmental stages in bitter and sweet apricot kernels to identify candidate genes involved in prunasin and amygdalin metabolism. We provide insight into the genetics associated with the molecular regulation of apricot kernel taste.

## Materials and methods

### Plant material

The *P. armeniaca* × *P. sibirica* (kernel consumption apricot, 'Youyi', YY, sweet kernel) and *P. sibirica* ('Aohanqi-39', AO, bitter kernel) were grown at the experimental farm of the Research Institute of Non-timber Forestry in Yuanyang County, Henan Province, China. The two apricot species show the same stages of fruit development, kernels representing six different developmental stages were collected throughout the complete kernel development between days 15–90 after flowering (between the beginning of kernel expansion to kernel maturation), which included 15 DAF (S1), 30 DAF (S2), 45 DAF (S3), 60 DAF (S4), 75 DAF (S5), and 90 DAF (S6). A total of 24 samples, including 12 bitter and 12 sweet kernel species, were collected. Three replicates for each sample were collected. All samples were frozen in liquid nitrogen and stored at −80 °C until further analysis.

### Determination of prunasin and amygdalin content

High-performance liquid chromatography (HPLC) was used to determine the prunasin and amygdalin content in the kernels, based on the method of the grain industry standard in China for grain and oil inspection^[24]^. The kernels were ground into powder and 0.4 g of kernel flour was defatted with 30 ml of petroleum benzine using a Soxhlet extractor. The degreased powder was added to 20 ml of methanol. The suspension was sonicated in an ultrasonic generator for 30 min. The methanol extract was filtered into another centrifuge tube using qualitative filter paper and 1.0 ml of filtrate was collected, combined with 1.0 ml of 20% methanol, diluted, and mixed. After filtration through a microporous filter to clarify the solution, it was transferred to a sample bottle for analysis. HPLC analysis was performed on an Agilent 1260 system with a Hypersil C18 column (250.0 mm × 4.6 mm, 5 μm) at 30 °C. The mobile phase was methanol : water = 20 : 80 (V : V), with a flow rate of 1.0 mL/min, a detection wavelength of 210 nm, and an injection volume of 10 μL. Linear regression was used to quantify the concentration of prunasin and amygdalin based on a standard curve equation of the standard peak area to concentration. SPSS 23 software was used to analyze the correlation and significance of the prunasin and amygdalin content.

### Transcriptome sequencing (RNA-seq)

We performed RNA-seq on samples from six different developmental stages of AO and YY. Total RNA from bitter and sweet kernel samples were extracted, purified, and analyzed for quality. Next, cDNA libraries were constructed and sequenced to generate 2 × 150 bp paired-end sequencing (PE150) on an Illumina Novaseq™ 6000 (LC-Bio Technology Co., Ltd., Hangzhou, China) following the manufacturer's protocol. Low-quality reads were removed using fastp software with default parameters (https://github.com/OpenGene/fastp) and Illumina sequencing reads were mapped to a reference genome (cultivar 'F106', Genome Database for Rosaceae, tfGDR1049).

### Transcriptome data analysis

The mapped reads for each sample were assembled using StringTie with default parameters (https://ccb.jhu.edu/software/stringtie). StringTie was used to estimate the gene expression levels by calculating FPKM^[25]^. The significant differentially expressed genes (DEGs) were selected with a |Log2fold-change| > 2 and a q value < 0.05 by edgeR. A Kyoto Encyclopedia of Genes and Genomes (KEGG) pathway enrichment analysis and functional annotation were performed and the number of DEGs enriched for each term was determined. We performed principal component analysis (PCA) on the DEGs and a short time-series expression miner (STEM)^[26]^ analysis on AO and YY, respectively, both using the OmicShare tool platform (www.omicshare.com). The transcription factors (TFs) were identified using PlantTFDB (http://planttfdb.gao-lab.org/help_famschema.php).

### WGCNA and visualization of gene networks

DEGs with a FPKM > 1 in at least one sample were screened, which were used to perform a weighted gene co-expression network analysis (WGCNA) using the WGCNA package in R. Modules. The default settings included a soft power of 26, a minimum module size of 30, and a merge cut height of 0.3. Mining potential candidate DEGs in the amygdalin metabolism pathway in the module was done using the highest phenotypic relevance. Hub genes with potentially important functions were screened based on correlation coefficients (|MM| > 0.7), gene significance (|GS| > 0.7), and the degree of connectivity. Gene networks were visualized based on the WGCNA modules using Cytoscape 3.10.1 software.

### Validation of DEGs by qRT-PCR

Total RNA from each sample was reverse-transcribed using the All-in-One First-Strand Synthesis MasterMix kit (Thermo Scientific, Waltham, MA, USA). Specific primers of five candidate DEGs were designed using Primer Premier 5 (PREMIER, Palo Alto, CA, USA) software (Supplemental Table S1). *UBQ1* was used as an internal control to normalize the qRT-PCR results. The reactions were performed using the 2×SYBR Green qPCR Premix (Beijing Codon Company, Kemix) kit. Each reaction was repeated three times and the relative expression of the target gene was calculated using the 2^−ΔΔCᴛ^ method^[27]^. Pearson correlation coefficients between the fold-change between qRT-PCR and RNA-seq were calculated using SPSS 23 software.

## Results

### Prunasin and amygdalin content

**Figure 1:**
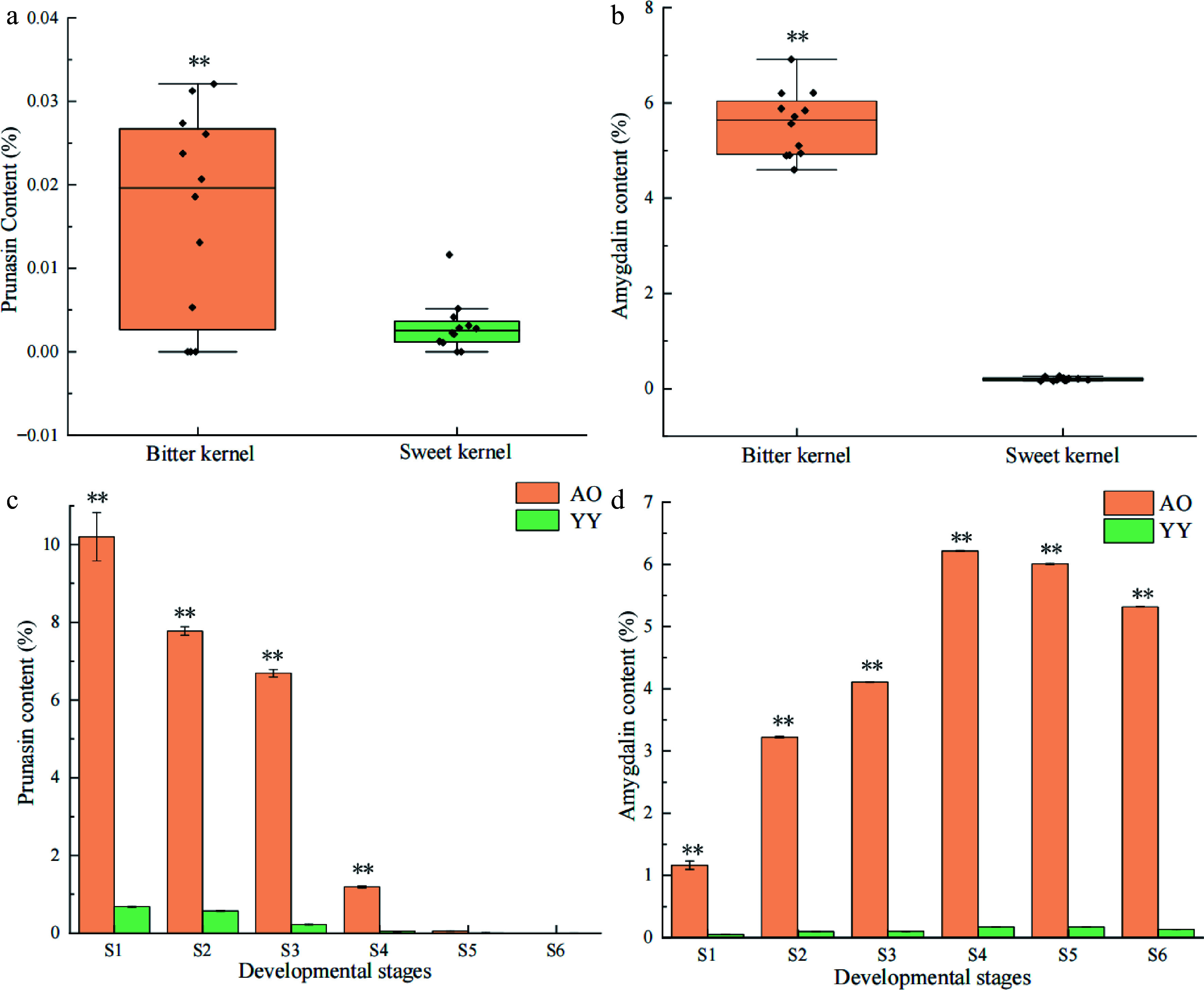
Patterns in the content of prunasin and amygdalin in *P. sibirica* and kernel consumption apricot*.* Significance testing was conducted using a T-test (* *p* < 0.05, ** *p* < 0.01), three replicates for each sample*.* (a) Prunasin content of bitter and sweet kernels at the mature stage. (b) Amygdalin content of bitter and sweet kernels at the mature stage. (c) Prunasin content at different developmental stages (S1–S6) of bitter and sweet kernels. (d) Amygdalin content at different developmental stages (S1–S6) of bitter and sweet kernels.

We measured prunasin and amygdalin content in the kernels of 24 varieties of apricots (Supplemental Table S2). The results indicated that the prunasin and amygdalin content in the bitter kernels was significantly higher compared with that of sweet kernels (Fig. 1a, b). To identify the basis for the differences in amygdalin levels in bitter versus sweet kernels, we extracted and measured prunasin and amygdalin in the bitter (AO) and sweet kernels (YY) at different developmental stages. The results indicated that prunasin and amygdalin content in AO was higher compared with that in YY at six different developmental stages (Fig. 1c & d). In both AO and YY, prunasin exhibited a decreasing trend during kernel development and the content in AO decreased at the greatest rate during the S3–S4 stage (82.21%). For YY, prunasin content decreased at the greatest rate during the S2–S3 stage (59.65%). In both AO and YY, amygdalin content exhibited a continuous increase from S1 to S4 and a decrease from S5 to S6 stage. In AO, amygdalin content increased sharply from S3 to S4, reaching a peak (6.22%) in S4, whereas it was lower in YY (< 1.00%) at six different development stages, and reaching a peak (0.18%) in S4.

The correlation between prunasin and amygdalin content at six different developmental stages was analyzed. The results indicated that prunasin and amygdalin content was significantly negatively correlated in both bitter and sweet kernels with correlation coefficients of −0.94 and −0.89, respectively.

### RNA-seq analysis

To determine the regulatory mechanism in apricots, we performed RNA sequencing (RNA-seq) on bitter and sweet kernels at six different developmental stages. A total of 266.76 Gb of clean data were obtained with an average of 7.41 Gb. The average Q30 was 98.47% and the GC content accounted for 47.61% (Supplemental Table S3). An average of 94.44% high-quality reads were mapped to the reference genome. The results indicated that the data quality was robust and could be used for further analysis.

**Figure 2:**
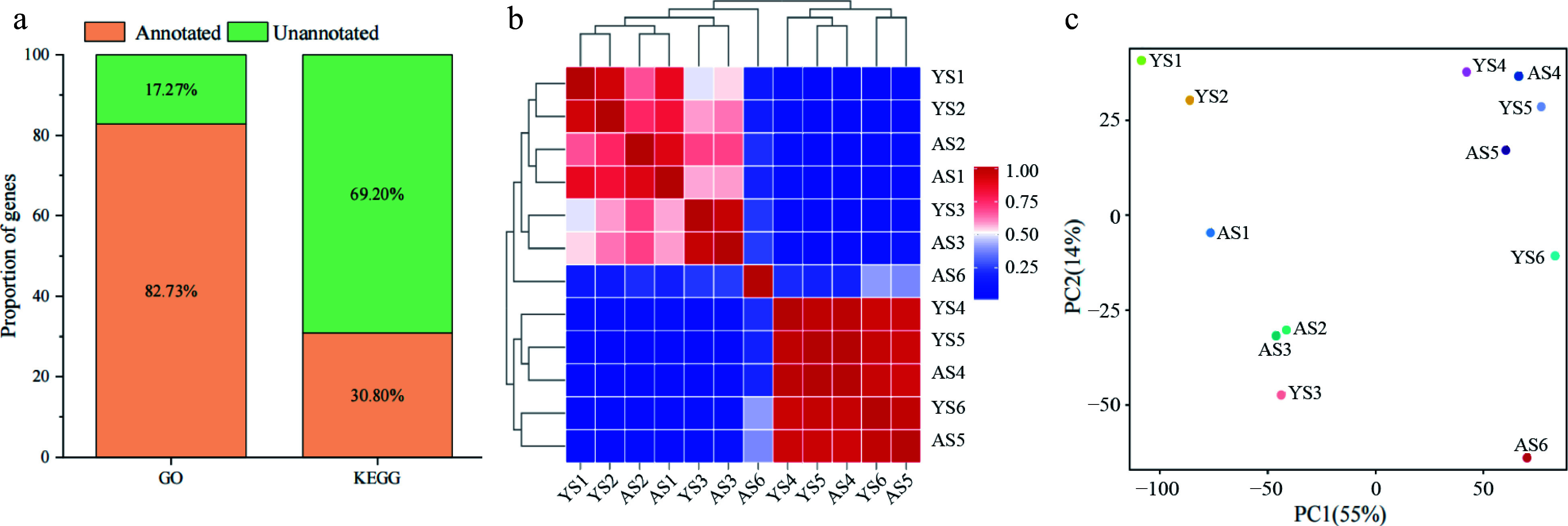
Analysis of gene in the transcriptome of bitter and sweet kernels. (a) Annotation of DEGs in GO and KEGG databases. (b) Pearson correlation coefficient (PCC) at six different developmental stages of 'Aohanqi-39' (AO, AS1–AS6) and 'Youyi' (YY, YS1–YS6). (c) Principal Component Analysis (PCA) at six developmental stages of 'Aohanqi-39' (AO) and 'Youyi' (YY).

A total of 27,062 genes were expressed in at least one of the 36 samples and the three replicates for each sample exhibited a high Pearson correlation coefficient (PCC) (0.92–1.00) (Supplemental Fig. S1a & b), indicating the high quality of the replicates for each tissue sample. The expressed genes of two apricot species in similar proportions at different stage, and the proportions of the gene distribution for the four expression levels were essentially similar, with the highest proportion of the genes exhibiting an expression of 0 ≤ FPKM < 1 and ranging from 50.22% to 65.50%, and the lowest proportion of the genes with a high expression of FPKM ≥ 50, ranging from 2.75% to 7.78% (Supplemental Fig. S2). Next, to further investigate the functional annotation of the expressed genes, we compared the genes with two databases, GO and KEGG, and the results revealed that the proportion of genes annotated to the both databases was 82.73% and 30.80%, respectively (Fig. 2a).

To analyze the transcriptome of the bitter and sweet kernels at different stages, we performed a PCA and PCC analysis based on 27,062 expressed genes (Fig. 2b & c). The results indicated that YY as a whole could be divided into two groups, S1–S3 and S4–S6, whereas AO could be divided into three groups, S1–S3, S4–S5, and S6 respectively. Samples of AO and YY exhibited a low correlation during S3 and S4 stages, S4–S6 of YY and S4–S5 of AO had a high correlation, but AS6 showed a lower correlation with the other samples. Taken together, the results suggest that AO and YY have different transcriptional mechanisms.

### Differentially expressed gene analysis of amygdalin accumulation in bitter and sweet kernels

**Figure 3:**
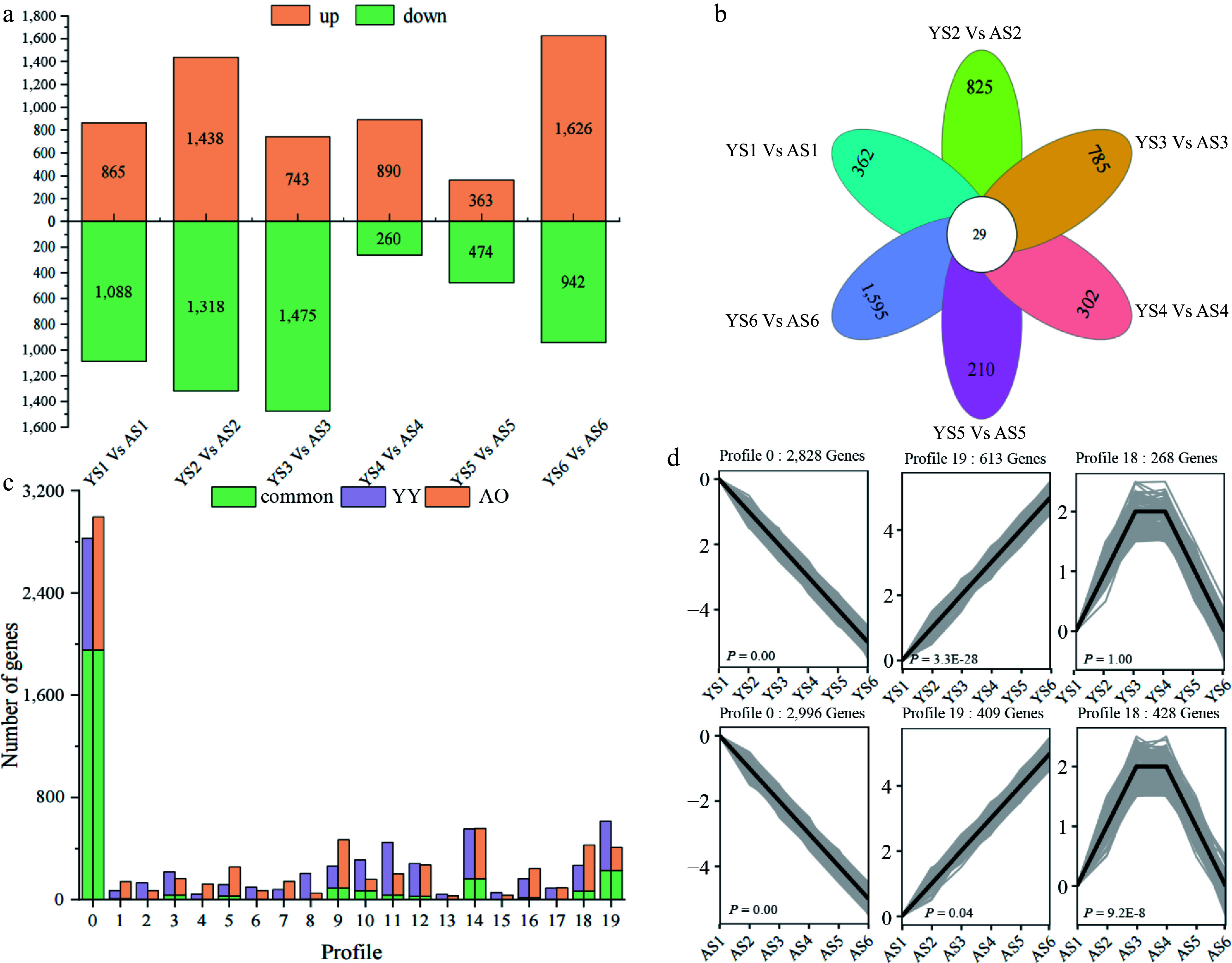
Differences in the number of DEGs as well as expression patterns between 'Youyi' (YY) and 'Aohanqi-39' (AO) at different developmental stages. (a) The number of up-regulated (yellow bars) and down-regulated (green bars) genes at different developmental stages in YY and AO. (b) The number of common and unique DEGs at different developmental stages in YY and AO. (c) The clustering profiles of the expression trends of all DEGs using STEM analysis.

To identify DEGs associated with regulatory mechanisms of bitter and sweet kernels in apricots, we selected 6,942 DEGs for comparison at the same stage in AO and YY. Overall, 260 to 1,626 DEGs were found to be down-regulated in the six stages between YY and AO (Fig. 3a). The highest number of DEGs was found in YS2 vs AS2 (2,756), followed by YS6 vs AS6 (2,568), and the lowest number of DEGs was found in YS5 vs AS5 (837). Only 29 DEGs were co-expressed between the six comparator groups, and 362, 825, 785, 302, 210, and 1,595 DEGs were uniquely differentially expressed in YS1 vs AS1, YS2 vs AS2, YS3 vs AS3, YS4 vs AS4, YS5 vs AS5, and YS6 vs AS6, respectively (Fig. 3b). We hypothesize that the varying numbers in DEGs between YY and AO may be related to the higher amygdalin content in the bitter kernels of *P. sibirica*.

To further analyze the expression of DEGs in sweet and bitter kernels at different stages, a short time-series expression miner (STEM) analysis was performed using all DEGs, of which 6,876 and 6,904 genes were clustered in YY and AO, respectively. Both were clustered into 20 different expression patterns (profile 0–19) (Supplemental Fig. S3). The analysis revealed that the number of DEGs differed in the different profiles of bitter and sweet kernels. Based on our analysis, we identified six profiles related to the prunasin and amygdalin content (Fig. 3c & d). The results indicated that profile 0 exhibited a decreasing trend in both AO and YY, consistent with the trend in the prunasin content. Profile 0 contained 2,996 DEGs in AO with 190 TFs, including *MYB* (31), *ERF* (25) and *bHLH* (24). In YY, there were 2,828 DEGs with 158 TFs in profile 0, including *MYB* (29) and *bHLH* (20). A total of 1,953 DEGs were co-expressed in AO and YY with 79 TFs, including MYB (14) and *bHLH* (14) families. Profile 19 exhibited an upward trend in both AO and YY, which was opposite to the trend observed for prunasin content. Profile 19 had a total of 409 DEGs in AO with 24 TFs, which were distributed in 19 different families. There were 613 DEGs in YY, including 46 TFs. Profile 19 comprised 228 co-expressed DEGs in AO and YY with only seven TFs. Profile 18 exhibited a trend of increasing first and then decreasing in AO and YY, which was consistent with the pattern of amygdalin content. Profile18 had a total of 428 DEGs in AO, including 26 TFs from 15 families. There were 268 DEGs in YY, 11 of which were TFs. Only one TF (*NF-YB*) in the 65 DEGs was shared by AO and YY. Among them, the expression patterns of *ERF* (PaF106G0100004607.01), *B3* (PaF106G0700027391.01), and *bHLH* (PaF106G0500020042.01) displayed a decreasing trend throughout the developmental stage, consistent with the trend of the prunasin content. Furthermore, PaF106G0100004607.01 showed higher expression in YY than in AO from S1 to S3; PaF106G0500020042.01 showed higher expression in AO than in YY from S1 to S2. Theses DEGs may play an important role in the transcriptional regulation of prunasin and amygdalin metabolism in the apricot kernel.

### Identification of DEGs in the synthesis, degradation, and detoxification pathways of bitter amygdalin

**Figure 4:**
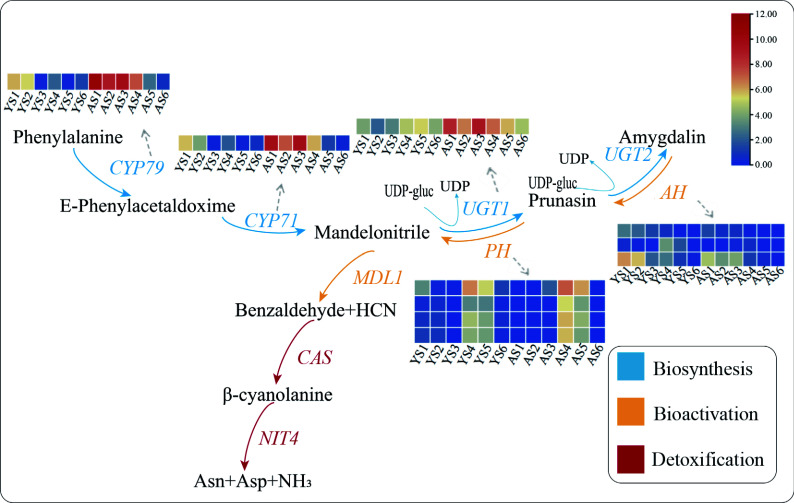
Expression patterns of relevant DEGs in the cyanoamino acid metabolic pathway. Red indicates high expression and blue indicates low expression. The IDs of the DEGs in the pathway were showed as following, *CYP79*: PaF106G0600021916.01; *CYP71*: PaF106G0500020435.01; *UGT1*: PaF106G0100005987.01; *AH*: PaF106G0200008023.01, PaF106G0600023212.01, and PaF106G0400014231.01; *PH*: PaF106G0600021586.01, PaF106G0600022768.01, PaF106G0600022773.01, and PaF106G0600022775.01.

To screen for key genes involved in the molecular regulation of bitter and sweet kernels of apricots, we performed a KEGG enrichment analysis of the DEGs. A total of 2,171 DEGs were subject to KEGG pathway analysis, of which 38 were enriched in the cyanoamino acid metabolic pathway (Supplemental Figs S4 & S5). Furthermore, we combined the expression patterns of each gene in the cyanoamino acid metabolic pathway with the prunasin and amygdalin content to identify candidate genes associated with the accumulation of amygdalin (Fig. 4).

In the biosynthesis pathway, the expression of *CYP79*, *CYP71,* and *UGT1* were much higher in AO than in YY at different developmental stages, consistent with the finding that the content of amygdalin were higher in bitter kernels than that in sweet kermels, including these genes may positively regulate amygdalin biosynthesis. Two *CYP79s* (PaF106G0600021916.01 and PaF106G0700027595.01) were identified and the expression of PaF106G0600021916.0 was consistent with the trend in prunasin content. A total of seven *CYP71s* were identified (PaF106G0300012392.01, PaF106G0300012395.01, PaF106G0300012396.01, PaF106G0500020430.01, PaF106G0500020431.01, PaF106G0500020434.01, and PaF106G0500020435.01). The expression pattern of PaF106G0500020435.01 in YY was consisted with the change in prunasin content. Two *UGT1s* (PaF106G0100005987.01 and PaF106G0200007937.01) were identified, and PaF106G0100005987.01 exhibited a pattern of up-regulation initially and then down-regulation in YY, which followed the same trend as the change in amygdalin content. *UGT2* was barely expressed in the bitter or sweet kernels.

In the bioactivation pathway, 18 *AH*s were identified (PaF106G0100000202.01, PaF106G0100003396.01, PaF106G0200008023.01, PaF106G0300014231.01, PaF106G0400015769.01, PaF106G0400015877.01, PaF106G0400015975.01, PaF106G0400015976.01, PaF106G0400018172.01, PaF106G0500021203.01, PaF106G0600021582.01, PaF106G0600022416.01, PaF106G0600023044.01, PaF106G0600023129.01, PaF106G0600023212.01, PaF106G0700027870.01, PaF106G0700028146.01, and PaF106G0800030614.01). We found that the expression of PaF106G0200008023.01, PaF106G0600023212.01, and PaF106G0400014231.01 in sweet kernels was higher compared with that in bitter kernels. Of these, the expression patterns of PaF106G0200008023.01 and PaF106G0600023212.01 in AO and YY were the same as the trend in prunasin content, indicating that these two genes are key DEGs that regulate the biosynthesis of prunasin and amygdalin. A total of four *PHs* (PaF106G0600021586.01, PaF106G0600022768.01, PaF106G0600022773.01, PaF106G0600022775.01) were identified, which were higher expressed in AO than that in YY, and the expression pattern was the same as the pattern observed for amygdalin content. Two *MDLs* (PaF106G0700027442.01 and PaF106G0700027452.01) were identified, however, their expression did not show a significant difference between AO and YY at the same stage.

In the detoxification pathway, *CAS* and *NIT4* are primarily involved in the detoxification of amygdalin metabolites (HCN). Two *CASs* (PaF106G0200009036.01, PaF106G0600025563.01) and one *NIT4* (PaF106G0700027936.01) were identified. PaF106G0600025563.01 and PaF106G0700027936.01 exhibited an overall up-regulated expression trend, and PaF106G0200009036.01 showed an upward trend at early stages and a downward trend at the late stages, however, the expression pattern of these genes were not consisted with the content of prunasin and amygdalin.

### Co-expression networks of genes associated with kernel bitter and sweet

**Figure 5:**
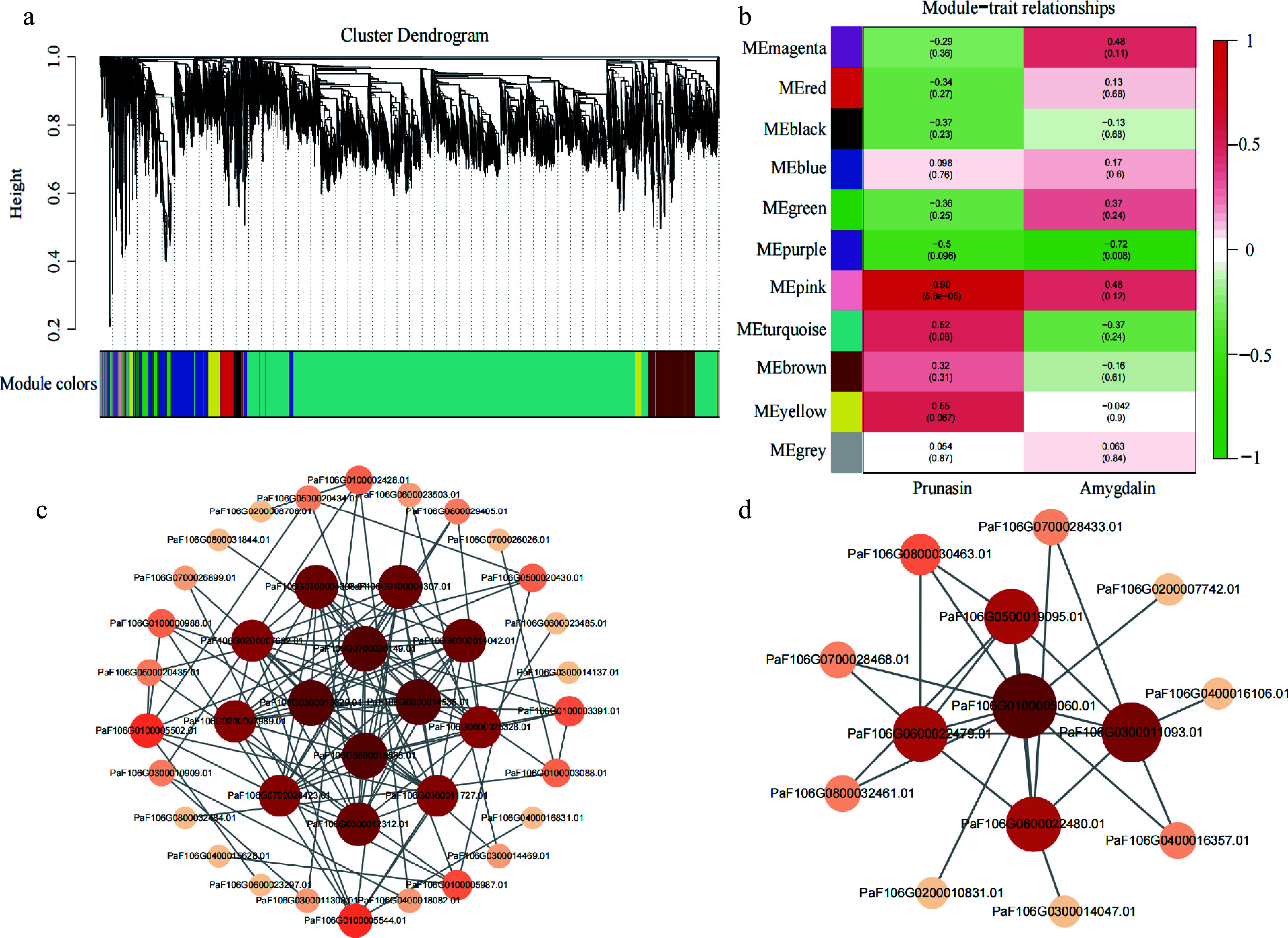
Identification of hub genes in a co-expression network. (a) Hierarchical clustering dendrogram showing 11 co-expression gene modules by WGCNA. (b) Correlations and corresponding *p*-values of different modules with prunasin and amygdalin content. (c) Co-expression network of 22 genes in the pink module. (d) Co-expression network of 10 genes in the purple module.

To determine the regulatory network of genes associated with the bitter-sweet trait of apricot kernels, we performed WGCNA. Correlations between the expression pattern of DEGs and the trends of prunasin and amygdalin content at different developmental stage were established and 11 modules were identified (Fig. 5a). The results indicated that the correlation coefficient of the pink module with prunasin content was the highest (r = 0.90, *P* = 5.6e-05), whereas the purple module with amygdalin content was the highest (r = −0.72, *P* = 0.008) (Fig. 5b). This indicated that the two modules had the greatest relevance to the bitter trait of apricot kernels.

Further analysis revealed 66 DEGs, including two TFs, that were identified in the pink module, then, we selected 22 DEGs as candidate genes (Supplemental Table S4) (two TFs, two cyanoamino acid metabolism genes, and other structural genes or regulators) based on module membership (MM), gene significance (GS), and weight values (top 100), and constructed a co-expression network (Fig. 5c). And then, based on the degree of connectivity between candidate genes, we identified four key hub genes (PaF106G0700026149.01, PaF106G0300013629.01, PaF106G0500018585.01, and PaF106G0300014535.01), in which the expression patterns were higher in bitter kernels compared with that in sweet kernels. These four genes were considered candidate genes associated with prunasin content and positively regulate prunasin biosynthesis.

In the purple module, 44 genes were significantly negatively correlated with amygdalin content, and 10 candidate genes were selected to construct a gene co-expression network (Supplemental Table S4, Fig. 5d). One key hub gene was identified (PaF106G0100005060.01), in which its expression was higher in sweet kernels compared with that in bitter kernels. The results suggest that this gene negatively regulates amygdalin biosynthesis.

### qRT-PCR validation of DEGs

**Figure 6:**
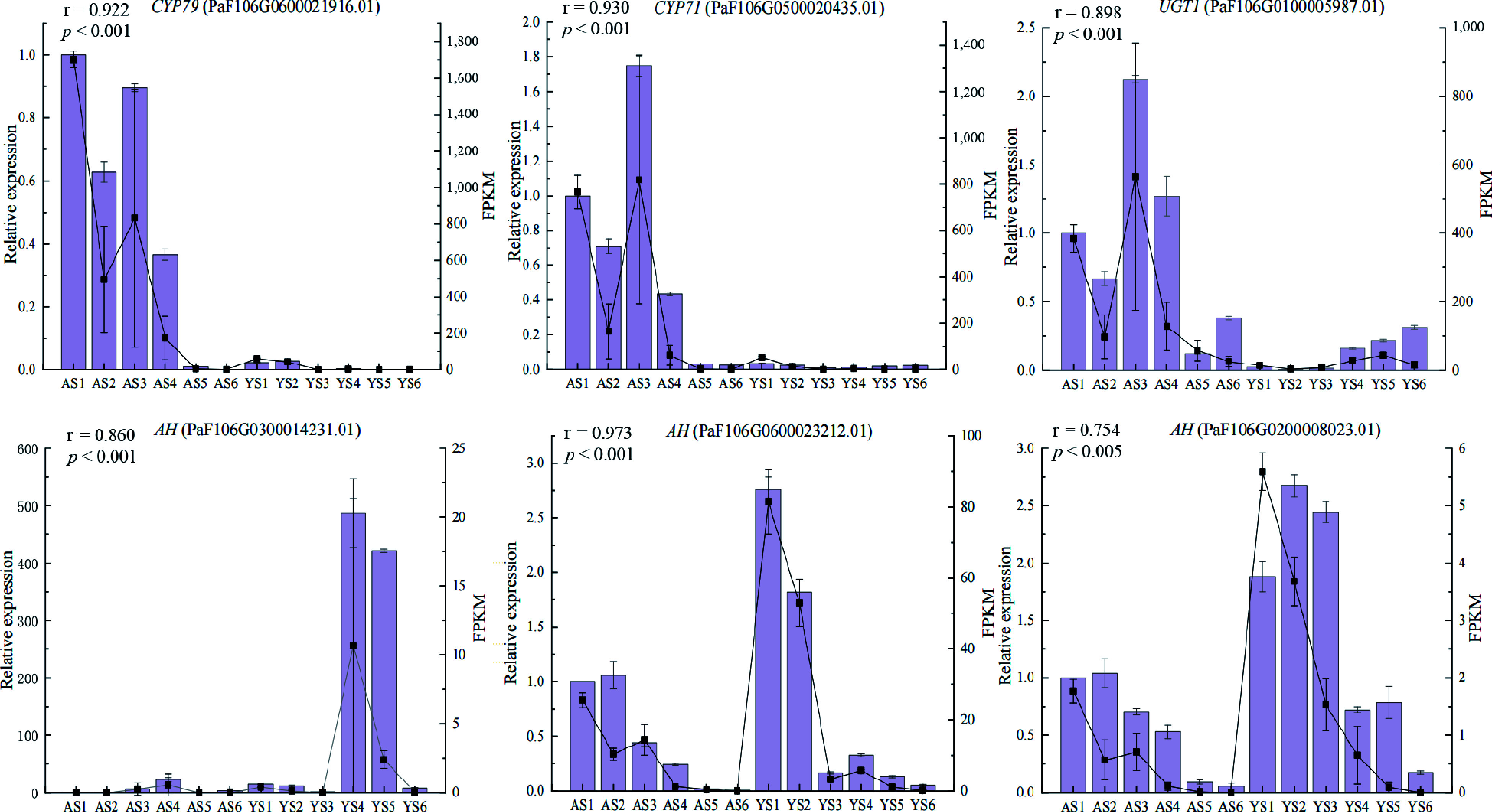
Expression levels of six candidate DEGs validated by qRT-PCR. Columns and lines indicate qRT-PCR and RNA-seq of the candidate DEGs, respectively. Pearson correlation coefficients were calculated between qRT-PCR and RNA-seq data of candidate DEGs.

To further assess the validity of the RNA-seq data, qRT-PCR was done to validate the relative expression of six candidate DEGs (Fig. 6). The results indicated that the qRT-PCR and RNA-seq data of DEGs exhibited a significant correlation (*p* ≤ 0.05, r > 0.75), which supported the reliability of the RNA-seq data.

## Discussion

### The change rule of cyanoside content

In this study, we determined the prunasin and amygdalin content in the kernels of 24 apricot varieties. The results indicated that the content of both in bitter kernels was significantly higher compared with that in sweet kernels (Fig. 1a & b), which is consistent with the results of previous studies^[5,12,28]^. Next, we measured prunasin and amygdalin content in bitter and sweet kernels at six different developmental stages. The results indicated that prunasin exhibited a decreasing trend with the maturation of kernels for both bitter and sweet kernels, and amygdalin showed an increasing and then decreasing trend in both kernels (Fig. 1c & d), which was different from the previous reports^[29]^. The content of amygdalin showed varies in different plants^[30]^, and prunasin and amygdalin content in apricot kernels are may affected by apricot species and growing environment. Prunasin is a precursor substance of amygdalin and a negative correlation was observed between prunasin and amygdalin^[29]^, which is consistent with our study, indicating the accumulation of cyanogenic glycosides during kernel development was dominated by the transition from prunasin to amygdalin.

### Metabolic pathway of amygdalin

Prunasin and amygdalin are cyanogenic glycosides, which are biologically active plant products derived from amino acids. There are more than 3,000 plant species that contain cyanogenic glycosides^[31]^. The process of amygdalin metabolism has been proposed in* P. dulcis* and *Prunus amygdalus*. However, the regulatory mechanism of the bitter–sweet trait in apricot kernels remains unclear. In the present study, we examined the expression pattern of DEGs between bitter and sweet kernels at different developmental stages and identified 10 candidate DEGs associated with the metabolism of prunasin and amygdalin. Amygdalin biosynthesis is primarily catalyzed by *CYPs* and glucosyltransferase (*UGTs*)^[32,33]^. *CYPs* were expressed very low or no expression in *Prunus dulcis* (sweet kernels), but were expressed higher in bitter kernels of *Prunus mume*^[34,35]^. These results are consistent with that of our study, in which we identified *CYP79* (PaF106G0600021916.01) and *CYP71* (PaF106G0500020435.01) in bitter kernels with higher expression compared with that in sweet kernels. The expression pattern of these two DEGs in both bitter and sweet kernels was similar to the content of prunasin. In addition to *UGT1*^[36]^, *MDL1*^[37]^, *PH691*, and *PH692*^[38]^ were also identified in previous studies. Glycosyltransferase (*UGT*) also plays an important role in the metabolism of amygdalin as catalyzes the formation of amygdalin^[34,36,39]^. In *P. dulcis*, the activity of *UGT* is more than three-fold greater in the bitter kernels than that in the sweet kernels^[36]^. The expression pattern of *UGT1* (PaF106G0100005987.01) identified in this study was correlated with the pattern of amygdalin content, and the expression was higher in bitter kernels compared with sweet kernels, which was considered to be a key candidate gene for the amygdalin metabolism.

In the degradation pathway of amygdalin, the expression of bitterness traits in the kernel may be associated with the lack of metabolic mechanisms related to β-glucosidase, which leads to the accumulation of prunasin and amygdalin, resulting in bitterness^[17,40,41]^. In this study, we identified seven β-glucosidases (three *AH* and four *PH*). The expression patterns of these DEGs correlated with the changing patterns of prunasin and amygdalin content. The expression of *AH* (PaF106G0200008023.01, PaF106G0600023212.01, and PaF106G0400014231.01) expression was higher in sweet kernels than in bitter kernels, indicating that the low expression of *AH* in bitter kernels might be an important contributing to amygdalin accumulation. However, Deng et al.^[29]^ found that sweet and bitter kernels differed significantly in their amygdalin content, but β-glucosidase exhibited high activity in the development of both bitter and sweet kernels. They concluded that β-glucosidase was not important for amygdalin accumulation. The different results obtained may be related to the apricot species and the different developmental stages of apricot.

TFs play an important role in the metabolic regulation of plants^[39, 41]^. *bHLH* were key TFs involved in amygdalin biosynthesis^[42]^, which regulating the transcription of *CYP79* and *CYP71* and resulting sweet flavors in the almond^[43]^. We performed TF prediction of DEGs in six importent STEM profiles, and identified three TFs (*ERF*, *B3*, and *bHLH*), displaying expressions consistent with changes in prunasin content, which could highlight potentially important TFs in regulating prunasin and amygdalin metabolism.

## Conclusions

In this study, we measured and analyzed the prunasin and amygdalin content in kernels of *P. sibirica* and kernel consumption apricot. We also performed a transcriptome analysis of kernels during the different developmental stages. The results indicated that amygdalin content in bitter kernels was significantly higher than that in sweet kernels. And the *CYP79*, *CYP71*, *UGT1*, *AH*, and *PH* in the cyanoamino acid metabolic pathway were considered to be important regulators of the prunasin and amygdalin metabolism in apricot kernels. Five potential key hub genes were also identified as potential regulators. Our study provides a genetic theoretical basis for the regulation of the bitter-sweet trait in apricot kernels. These candidate genes could be used for developing molecular markers to improve the breeding in apricot kernel.

## SUPPLEMENTARY DATA

Supplementary data to this article can be found online.

## Data Availability

Raw transcriptome sequencing reads were deposited into the Sequence Read Archive (SRA) under accession ID PRJNA1025947.
