# Supplementary material for: Transcriptome analysis reveals genes associated with the bitter-sweet trait of apricot kernels
Source: For Res (Fayettev). 2024 Feb 29;4:e007. doi: 10.48130/forres-0024-0004 (PMC11524293; doi:10.48130/forres-0024-0004)
Supplement: Supplementary file 1 — Supplementary data to this article can be found online. [file forres-0024-0004-S1.zip › 10.48130_forres-0024-0004-Suppl-FigureS2.pdf]

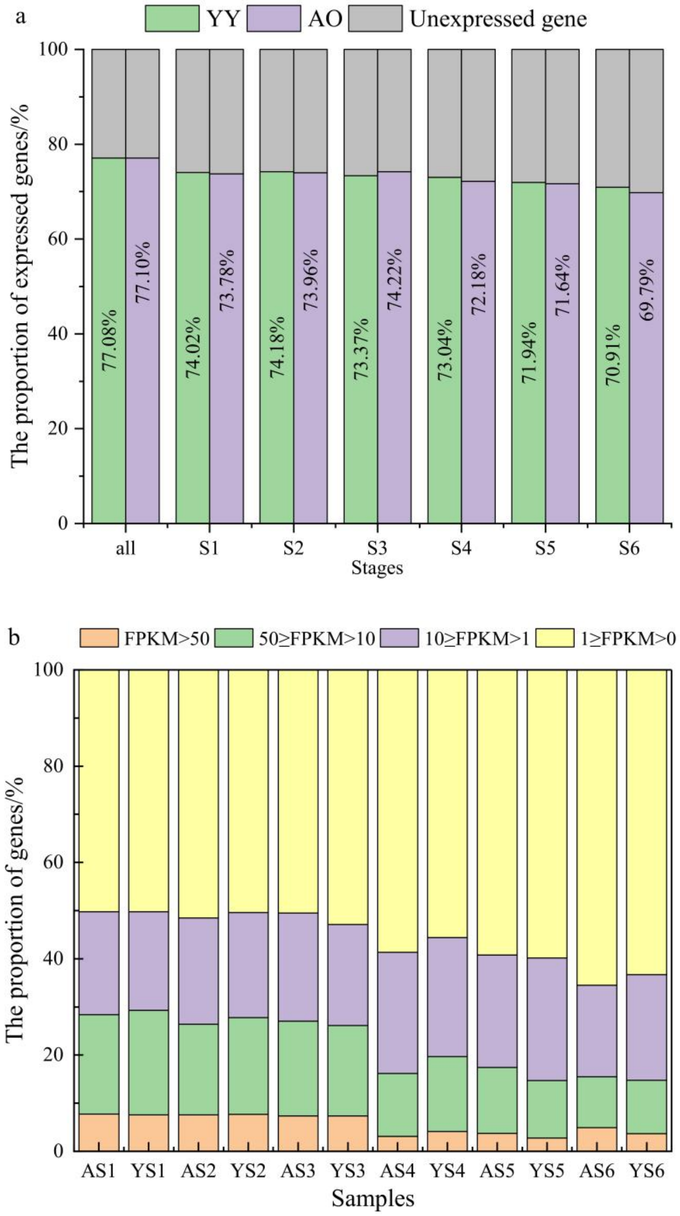

Supplemental S2. Analysis of gene expression in the transcriptome of bitter and sweet kernels at six different developmental stages. a. The proportions of expressed genes at total and different stages in "Youyi" (YY) and "Aohanqi-39" (AO). **b.**The proportion of genes with four different expression levels in "Aohanqi-39" (AO, AS1-AS6) and "Youyi" (YY, YS1-YS6).
