# Supplementary material for: Transcriptome analysis reveals genes associated with the bitter-sweet trait of apricot kernels
Source: For Res (Fayettev). 2024 Feb 29;4:e007. doi: 10.48130/forres-0024-0004 (PMC11524293; doi:10.48130/forres-0024-0004)
Supplement: Supplementary file 1 — Supplementary data to this article can be found online. [file forres-0024-0004-S1.zip › 10.48130_forres-0024-0004-Suppl-FigureS4.pdf]

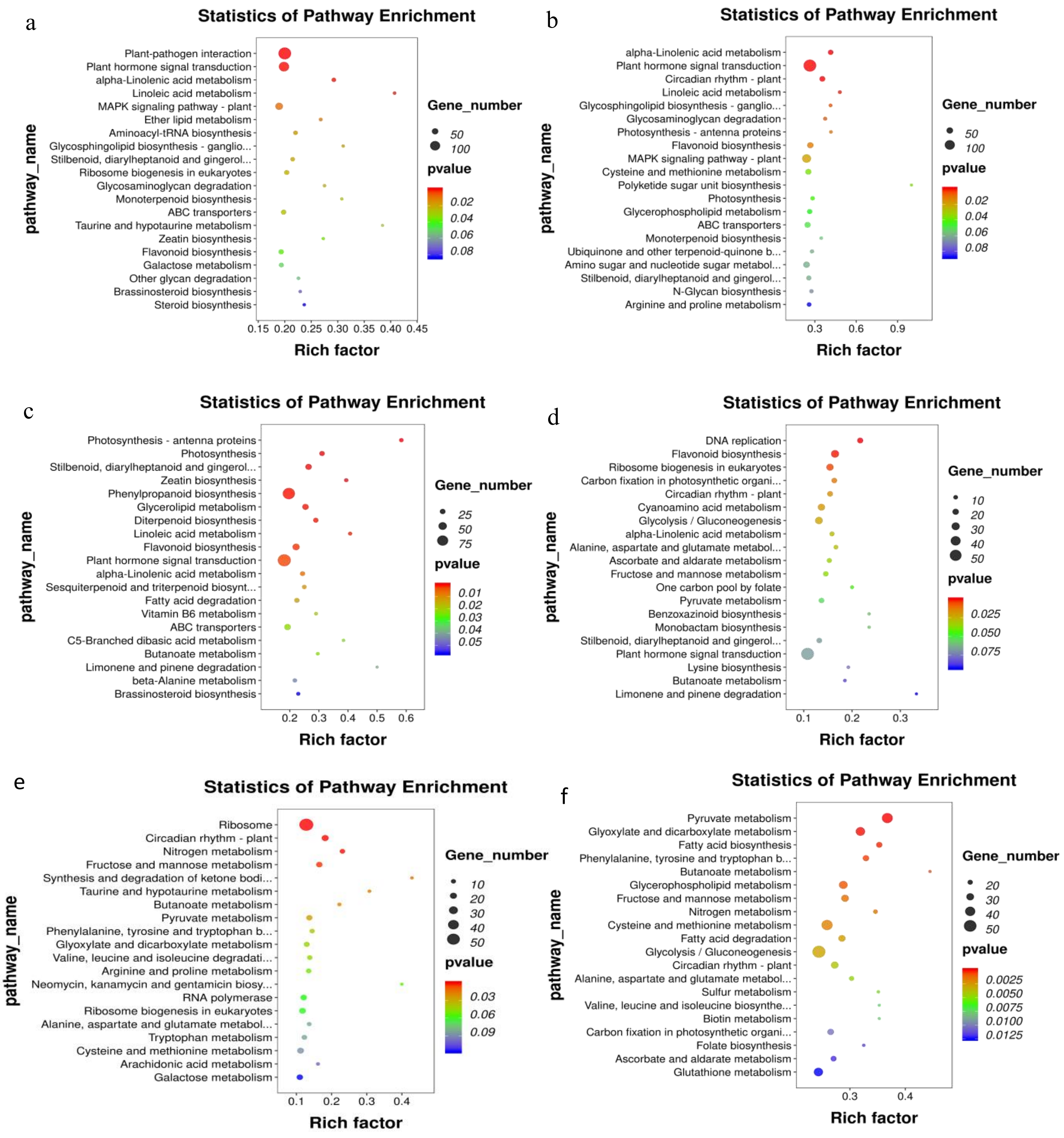

Supplemental Figure S4. KEGG pathway enrichment analysis of DEGs in the six different developmental stages comparison groups of “Youyi” (YY) and “Aohanqi-39” (AO). a~f were presented the KEGG enrichment of DEGs in stage of YS1 vs AS1 ~ YS6 vs AS6, respectively.
