# Supplementary material for: Transcriptome analysis reveals genes associated with the bitter-sweet trait of apricot kernels
Source: For Res (Fayettev). 2024 Feb 29;4:e007. doi: 10.48130/forres-0024-0004 (PMC11524293; doi:10.48130/forres-0024-0004)
Supplement: Supplementary file 1 — Supplementary data to this article can be found online. [file forres-0024-0004-S1.zip › 10.48130_forres-0024-0004-Suppl-FigureS5.pdf]

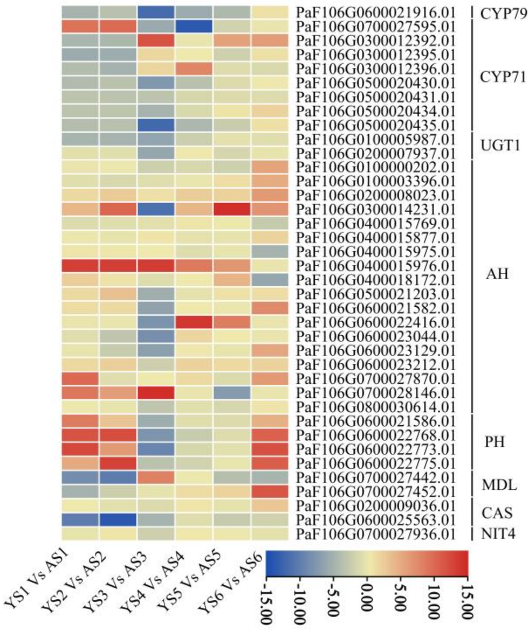

Supplemental Figure S5. Heatmap of the expression pattern of DEGs in the amygdalin metabolic pathway. Red indicates high expression and blue indicates low expression.
