# Supplementary material for: Transcriptome analysis reveals genes associated with the bitter-sweet trait of apricot kernels
Source: For Res (Fayettev). 2024 Feb 29;4:e007. doi: 10.48130/forres-0024-0004 (PMC11524293; doi:10.48130/forres-0024-0004)
Supplement: Supplementary file 1 — Supplementary data to this article can be found online. [file forres-0024-0004-S1.zip › 10.48130_forres-0024-0004-Suppl-FigureS1.pdf]

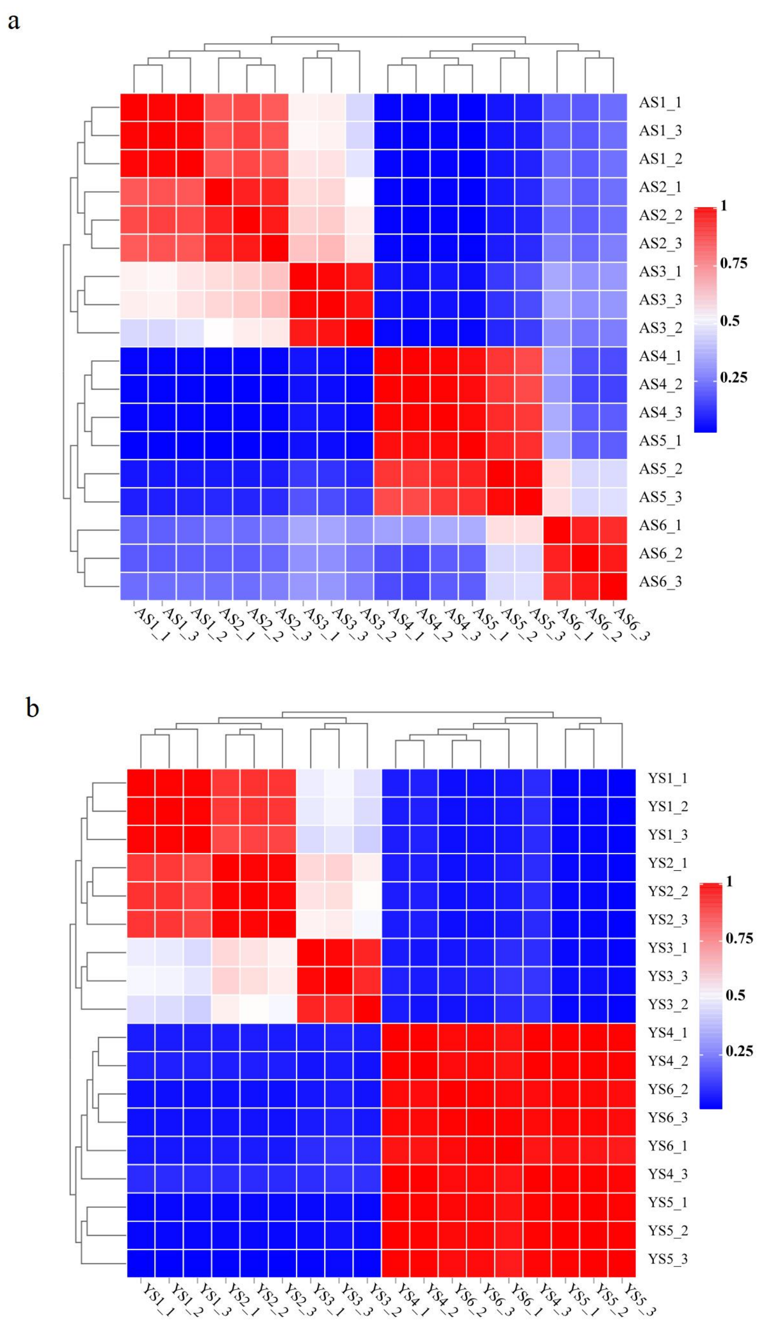

Supplemental Figure S1. Pearson correlation coefficient (PCC) analysis of gene expression data from the three replicate samples from each stage in bitter kernels ("Aohanqi-39" (AO, AS1~AS6)) and sweet kernels ("Youyi" (YY, YS1~YS6)). a. Pearson correlation coefficient (PCC) among the three biological replicates of six developmental stages in AO (AS1-AS6). **b.** PCC among the three biological replicates of six developmental stages in YY (YS1-YS6).
